# Association Between the Dietary Inflammatory Index and the Risk of Fracture in Chinese Adults: Longitudinal Study

**DOI:** 10.2196/43501

**Published:** 2023-08-17

**Authors:** Lu Wang, Chen Ye, Fanghong Zhao, Hongjing Wu, Ruoyu Wang, Zhaofeng Zhang, Jie Li

**Affiliations:** 1 Department of Epidemiology and Health Statistics Hebei Medical University Shijiazhuang China; 2 Department of Nutrition and Food Hygiene School of Public Health, Peking University Health Science Center Beijing China; 3 Beijing Center for Disease Control and Prevention Beijing China; 4 Beijing’s Key Laboratory of Food Safety Toxicology Research and Evaluation Beijing China; 5 Beijing Fengtai District Center for Disease Control and Prevention Beijing China

**Keywords:** dietary inflammatory index, fractures, diet, risk

## Abstract

**Background:**

Chronic inflammation plays a crucial role in tissue injury, osteoporosis, and fracture. The dietary inflammatory index (DII) is a tool for assessing the potential for inflammation in the diet. However, the association between the DII and fractures remains controversial from previous studies.

**Objective:**

We aimed to explore the correlation between the DII and fracture risk in Chinese adults.

**Methods:**

We included 11,999 adults (5519 men and 6480 women) who were a part of the China Health and Nutrition Survey (1997-2015) prospective cohort. A 3-day, 24-hour meal review method was used to calculate the DII score. The fractures were identified using a questionnaire. Cox proportional hazards models were used to estimate the hazard ratios (HRs) and 95% CIs for fractures. Subgroup, sensitivity, and restricted cubic spline analyses were performed.

**Results:**

During the 18 years of follow-up (median follow-up 9.0 years), 463 men and 439 women developed fractures. The median DII score was 0.64 (IQR −1.74 to 1.46) for the total sample, 0.75 (IQR −1.68 to 1.50) for men, and 0.53 (IQR −1.79 to 1.42) for women. The DII score had a positive correlation with the risk of fracture among women but not among men. For men, after adjusting for covariates, the HRs for quintiles of DII were 1, 0.96 (95% CI 0.66-1.41), 1.05 (95% CI 0.74-1.49), 0.89 (95% CI 0.62-1.26), and 0.94 (95% CI 0.67-1.34; trend: *P*=.62). The HRs for women were 1, 1.13 (95% CI 0.72-1.79), 1.24 (95% CI 0.83-1.86), 1.51 (95% CI 1.02-2.22), and 1.62 (95% CI 1.10-2.39; trend: *P*=.004). The restricted cubic spline analysis showed a significant association between fracture risk and DII score in women (overall association: *P*=.01); as the DII scores were >0.53, HRs showed a significant upward trend. Women aged <50 years or who are nonsmokers, who are nondrinkers, or with nonabdominal obesity had a positive association between fracture risk and the DII score. In sensitivity analyses, after excluding people with diabetes or hypertension, there was still a positive association between fracture risk and the DII score in women. Among the DII components, the DII scores of protein (trend: *P*=.03), niacin (trend: *P*=.002), and iron (trend: *P*=.02) showed significant associations with the risk of fracture in women.

**Conclusions:**

Proinflammatory diet consumption increased the fracture risk in Chinese women aged <50 years. The high consumption of anti-inflammatory foods and low consumption of proinflammatory foods may be an important strategy to prevent fractures in women.

## Introduction

### Fractures Hazard

Globally, fractures pose a serious economic burden and public health issue [1,2]. Fractures can result in work absence, decreased productivity, disability, impaired quality of life, health loss, and high health care costs and are a major burden on health care systems at large [3-5], especially in people with osteoporosis [6,7]. The aging of the population is associated with an increasing burden of fractures around the world [8]. A systematic review including 22 studies from high-income nations or regions and 3 from low- or intermediate-income nations indicated that hip fracture incidence still increased rapidly in low- or intermediate-income nations [9]. Hip fracture is the most devastating type of osteoporotic fracture. It is projected that the number of hip fractures around the world will increase from 1.26 million in 1990 to 4.5 million by 2050, about half of which are likely to occur in Asia, particularly in China [10]. China, the largest middle-income country, has the largest population in the world and has been experiencing unprecedented rapid population aging. Fractures will be a substantial health burden in China.

Fragility fractures caused by low-energy trauma (slips, trips, and falls from standing height) were studied extensively [11]. Fragility fractures are closely associated with bone density (osteopenia and osteoporosis) [12,13]. Osteoporosis is the leading cause of fragility fractures, with an osteoporotic fracture occurring every 3 seconds [14]. The study of Global Burden (1990-2019) indicated that China, with the high disease burden of disability-adjusted life years number in low bone mass–related fractures, ranks second in 204 countries and territories [15]. Injuries have recently been recognized in China’s long-term development agenda, *Healthy China 2030* [16]. Fractures are an important contributor to the injury burden in China. We need to provide specific fracture prevention strategies to support the development of the Healthy China policy.

### Fractures and Chronic Inflammation

Currently, the mechanism underlying osteoporosis and fractures remains unclear, but chronic inflammation is widely regarded as an important cause of osteoporosis. Several observational studies have examined the potential association between serum inflammatory markers, such as interleukin (IL)–6, tumor necrosis factor (TNF)–α, and C-reactive protein (CRP), and fractures [17-21]. Proinflammatory cytokines such as TNF-α and IL-6 in osteoclasts can lead to bone erosion [22,23]. Diet is an important source of inflammation [24]. Inflammation levels in the body can be mediated by dietary components [25-27], some nutrients (eg, saturated fatty acids and trans fatty acids) can increase inflammation levels, and some nutrients (eg, fiber and some vitamins) can decrease inflammation levels [23,28]. To quantify the inflammatory potential of diets in various population groups [24], Cavicchia [29] first proposed the dietary inflammatory index (DII) in 2009, and Shivappa [24] developed the evaluation indexes further in 2014. They included 6500 references; studied the effects of food parameters on 6 inflammatory markers (IL1-β, IL-4, IL-6, IL-10, TNF-α, and CRP); rated the proinflammatory, anti-inflammatory, and noninflammatory effects of each food parameter; and finally determined 45 dietary components to calculate DII. A higher DII score indicates that a diet is more likely to enhance the body’s inflammatory response, whereas a lower DII score indicates that the diet reduces inflammation levels in the body [28,30-32]. The DII is a good indicator to estimate dietary quality associated with inflammation and has also been used to explore relationships with various health outcomes, including hypertension, diabetes, hyperuricemia, metabolic syndrome, asthma, breast cancer, and colorectal cancer [33-39].

### Association Between the DII and Fractures

In recent years, several studies have been conducted to examine the association between the DII score and fractures [23,32,40-43]. However, the association between DII and fracture risk showed inconsistent results, which may be attributed to differences in age, gender, ethnicity, and time of observation. A study involving postmenopausal women reported that a high DII score was associated with an increased hip fracture risk [32]. Moreover, results from North American studies showed that a higher DII score was associated with a higher incidence of fractures [40]. However, these studies were limited to White women. In addition, in the Tasmanian Older Adult Cohort Study, a higher DII score was associated with lower bone density and an increased incidence of fractures in community-dwelling older men but decreased fracture incidence in women [41]. The Brazilian Osteoporosis Study showed a lack of association between DII and low-impact fractures in the Brazilian population [42]. These foreign studies, because of racial differences, are not specific to China. In addition, populations in Western countries tend to consume animal-based diets, whereas populations in Eastern countries, such as China, tend to consume plant-based diets, characterized by higher quantities of vegetables and fruits [44]. Depending on the diet, the level of inflammation also varies [29]. Results from participants based in the Guangdong province and Hong Kong cohort study showed that a proinflammatory diet was positively associated with hip fracture risk in men and women [23,43]. Because the participants in the 2 Chinese studies either were from an economically advanced region of China or had settled in the southern part of the country, we believe that these results are not representative of a typical Chinese diet [23,43].

Currently, the pattern of food consumption has changed with the rapid development of the Chinese society. Despite the improvement in diet quality, the current dietary pattern is still less than ideal. The diet in China is gradually being westernized, with an increased intake of red meat, processed meats, and sugary drinks, which are associated with higher levels of inflammation [45]. Currently, China proposed the suggestion of “reduced salt, reduced oil, reduced sugar, healthy oral cavity, healthy weight, and healthy bone (three reductions and three healthy),” which was the action of national health promotion. Therefore, this study, which prospectively examined the association between the DII and fracture risk in a large sample in China, aimed to provide theoretical support and guidance for the use of nutrition and diet to reduce the risk of fractures. We hope to use practical actions to help the Healthy China development agenda.

## Methods

### Study Population

**Figure 1:**
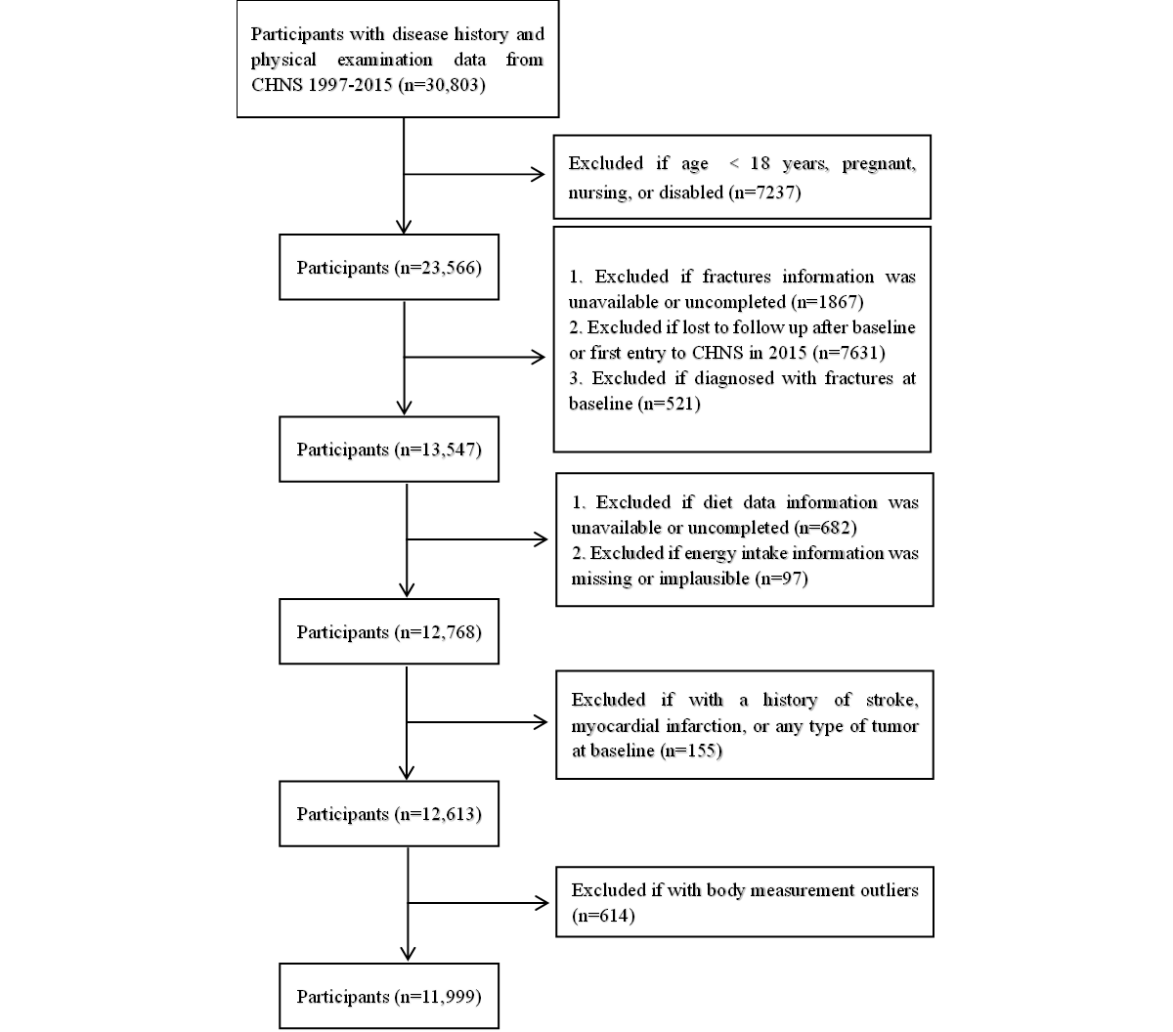
Study population flowchart. CHNS: China Health and Nutrition Survey.

This study used data from a subcohort of the China Health and Nutrition Survey (CHNS). Founded in 1989, the CHNS is a prospective cohort study that has conducted 10 follow-up surveys until 2015. In our study, we used the CHNS data obtained between 1997 and 2015. During this time, 30,803 participants with a disease history and physical examination data were enrolled. This study included adult participants aged ≥18 years. We excluded participants who were pregnant, nursing, or disabled and those without available or complete fracture data or follow-up data. We also excluded participants with missing or implausible energy intake information (>5000 or <700 kcal/d); a baseline diagnosis of fractures; or a history of myocardial infarction, stroke, or any type of tumor at baseline. We also excluded participants with body measurement outliers. Finally, we included 11,999 study participants, including 5519 men and 6480 women (Figure 1).

### Assessment of Nutrient Intake and the DII

Dietary intake assessment in the CHNS involved the 3-day, 24-hour meal review method for participating individuals and a household food inventory, which involved the weighing and measuring of products (used to obtain information on the consumption of edible oils and condiments) over the same 3 days (2 weekdays and 1 weekend day). The China Food Composition Tables Standard Edition provides general nutrition data for more than 3000 types of foods and ingredients in China. We combined the dietary data obtained from the 3-day, 24-hour meal review method with the China Food Composition Tables Standard Edition to obtain the data used to calculate the DII scores.

The mean intake of every food variable was transformed with standardized values from a world database into a *z* score, converted to a percentile, and centered by doubling and subtracting 1. Finally, the centered percentile score for each food variable was multiplied by its associated literature-derived inflammatory effect score and these scores were summed across the 45 dietary variables, thus providing an individual DII score. The higher the DII score, the more proinflammatory the diet; more negative values indicate more anti-inflammatory diets [24].

The energy-adjusted DII was calculated using the following steps, which were performed before the transformed *z* score. Energy adjustment was performed using the density method [46]; all the food variables and the world database were converted to units per 1000 kcal [47].

In total, 28 of the 45 possible food parameters were analyzed to obtain the overall DII scores. These included energy, carbohydrate, protein, total fat, alcohol, β-carotene, cholesterol, fiber, folic acid, niacin, iron, magnesium, selenium, zinc, monounsaturated fatty acids, polyunsaturated fatty acids, isoflavones, thiamine, riboflavin, saturated fat, vitamin A, vitamin C, garlic, ginger, onion, green or black tea, pepper, and thyme or oregano.

### Assessment of Covariates

On the basis of the Cox proportional hazards model of fractures, previous studies have analyzed demographic characteristics, lifestyle factors, physical measures, and disease information [41,48]. Information on demographic characteristics, obtained from a questionnaire, included age, gender, area of residence, marital status, level of educational attainment, and household income per capita (categorized into quartiles). Lifestyle factors obtained via a questionnaire included physical activity level (PAL; grouped into 3 levels), smoking status, and drinking status. Physical measures included the BMI, midarm muscle circumference (MAMC), and waist-to-hip ratio (WHR). The BMI was calculated as weight (kg) / height (m)^2^. MAMC was calculated as midupper arm circumference (cm) − π × (triceps skinfold thickness / 10). WHR was calculated as waist circumference (cm) divided by hip circumference (cm). According to the World Health Organization Asian WHR standard, a man with WHR ≥0.90 and a woman with WHR ≥0.85 are considered to have abdominal obesity [49]. The disease information including diabetes and hypertension was obtained using questionnaires. The Osteoporosis Self-assessment Tool for Asians (OSTA) index has been shown to distinguish between different degrees of osteoporosis. OSTA was calculated as 0.2 × (weight [kg] − age [years]); the following classification was used: OSTA <−4, high risk for osteoporosis and fracture; −4< OSTA <−1, medium risk for osteoporosis and fracture; and OSTA >−1, low risk for osteoporosis and fracture [50-52]. For all covariates, we used the baseline year measure.

### Outcome Identification

The outcome was defined based on the questionnaire survey. The participants were asked to report their history of fractures using a questionnaire-based interview at each follow-up since 1997. The questions were posed as follows: “(1) History of bone fracture? If yes, (2) age (years) at 1st bone fracture, and (3) number of times bone fracture?” We used the date when participants first entered the survey as the baseline date for participants according to the questionnaire. The follow-up person-time for each participant was calculated as the interval between baseline and the occurrence of fractures, the survey day in the last survey round before the participant was lost to follow-up, or the latest survey in 2015, whichever came first.

### Statistical Analysis

All statistical analyses were performed separately for men and women. We also divided the participants of each gender into 5 groups according to the quintiles of the DII. Participant baseline characteristics were described as a number (percentage) for categorical variables and means (SD) or as a median (IQR 25%-75%) for continuous variables. The chi-square test and rank-sum test were used to compare categorical and continuous variables between the men and women.

Cox proportional hazards models were used to estimate the hazard ratios (HRs) and 95% CIs for fractures. We also adjusted for multiple covariates before exploring the relationship between DII and fracture risk. To test the proportional hazards assumption, we conducted likelihood ratio tests. The results met the HR assumption. To calculate HRs among quintiles, the lowest intake quintiles were used as a reference. Three models were established. Model 1 was adjusted for demographic characteristics, including age, gender, residence area, highest education level, household income, and marital status. Model 2 was adjusted for lifestyle factors and physical measures: model 1+smoking status, drinking status, PAL, BMI, MAMC, and WHR. Model 3 adjusted for disease information: model 2+hypertension, diabetes, and OSTA index level. Tests for trends were performed for continuous variables using categorical DII scores by quintiles.

Stratified analyses were performed according to age, BMI, smoking status, drinking status, obesity status, OSTA index level, and MAMC. The *P* value for interaction was calculated with multiplicative terms by multiplying the quintiles of the DII by categorical variables used in the multivariable model.

Several sensitivity analyses were conducted in this study. Participants with hypertension or diabetes mellitus were excluded from the study. Participants were divided into 4 groups: nonhypertension, nondiabetes, nonhypertension and nondiabetes, as well as nonhypertension or nondiabetes.

Carbohydrate, total fat, protein, thiamine, riboflavin, niacin, folic acid, vitamin A, iron, selenium, zinc, and magnesium of the DII component were divided into 5 levels in men and women to estimate the HRs and 95% CIs for fractures. To calculate the DII scores for these nutrients, we controlled for energy consumption.

We used restricted cubic splines (RCSs) to test for linearity, and RCSs were used for DII in multivariable-adjusted Cox regression analyses (model 3) for women separately. The median values were used as references in the RCS analyses.

All statistical analyses were performed using SPSS (version 27.0; IBM Corp) and R (version 4.1.0; R Foundation for Statistical Computing). Two-sided *P* values <.05 were considered as statistically significant.

### Ethics Approval

The CHNS, is an international collaborative project between the Carolina Population Center at the University of North Carolina at Chapel Hill and the National Institute for Nutrition and Health (NINH, former National Institute of Nutrition and Food Safety) at the Chinese Center for Disease Control and Prevention (CCDC) with an ethical approval number of 2015017 [53].

### Participation and Informed Consent

The CHNS is an ongoing open cohort. All participants provided informed consent for inclusion before participating in the study and allowed for secondary analysis without additional consent. The study data are anonymized or deidentified.

## Results

### Baseline Characteristics

We included 11,999 adults (5519 men and 6480 women) who were part of the CHNS (1997-2015) prospective cohort. The average age of the participants was 44.0 (SD 14.6) years. Of the total 11,999 participants, 5519 (46%) were men and 6480 (54%) were women. The median DII score was 0.64 (IQR −1.74 to 1.46) for the total sample, 0.75 (IQR −1.68 to 1.50) for men, and 0.53 (IQR −1.79 to 1.42) for women. A histogram of the DII in men and women is presented in Figure S1 in Multimedia Appendix 1. During the 18 years of follow-up (median follow-up 9.0 years), we ascertained that 463 men and 439 women developed fractures. The baseline characteristics of the study population are described across the quintiles of DII in Table 1. The population baseline characteristics by gender are also presented in Table S1 in Multimedia Appendix 1. At baseline, marital status, education level, smoking status, drinking status, PAL, BMI, MAMC, WHR, abdominal obesity status, OSTA index level, carbohydrate, total fat, and protein levels were statistically different between men and women (all *P*<.001).

**Table 1:** Baseline characteristics of the study population in men and women^a^.

Characteristics			Qs^b^ of DII^c^ in men						Qs of DII in women				
			Q1 (n=1104)		Q3 (n=1104)		Q5 (n=1103)		Q1 (n=1296)		Q3 (n=1296)		Q5 (n=1296)
**Age (years), mean (SD)**			47.9 (14.0)		41.5 (14.5)		43.3 (14.3)		44.8 (14.8)		42.8 (13.9)		42.9 (14.0)
	<50	464 (42.03)		658 (59.6)		626 (56.75)		656 (50.62)		753 (58.1)		749 (57.79)	
	≥50	640 (57.97)		446 (40.4)		477 (43.25)		640 (49.38)		543 (41.9)		547 (42.21)	
**Marital status, n (%)**													
	Never married	79 (7.16)		178 (16.12)		140 (12.69)		44 (3.4)		60 (4.63)		84 (6.48)	
	Married	985 (89.22)		895 (81.07)		934 (84.68)		1138 (87.81)		1143 (88.19)		1123 (86.65)	
	Divorced	19 (1.72)		7 (0.63)		9 (0.82)		28 (2.16)		11 (0.85)		4 (0.31)	
	Widowed	21 (1.9)		24 (2.17)		20 (1.81)		86 (6.64)		82 (6.33)		85 (6.56)	
**Residence, n (%)**													
	Rural	419 (37.95)		815 (73.82)		733 (66.46)		554 (42.75)		958 (73.92)		880 (67.9)	
	Urban	685 (62.05)		289 (26.18)		370 (33.51)		742 (57.25)		338 (26.08)		416 (32.1)	
**Household income level (yuan ¥; yuan ¥1=US $0.14), n (%)**													
	Low (<8532)	254 (23.01)		299 (27.08)		285 (25.84)		308 (23.77)		365 (28.16)		339 (26.16)	
	Medium (8532-15,576)	235 (21.29)		308 (27.9)		305 (27.65)		293 (22.61)		330 (25.46)		329 (25.39)	
	High (15,577-30,500)	274 (24.82)		259 (23.46)		264 (23.93)		313 (24.15)		361 (27.85)		326 (25.15)	
	Very high (>30,500)	341 (30.89)		238 (21.56)		249 (22.57)		382 (29.48)		240 (18.52)		302 (23.3)	
**Education level, n (%)**													
	None	63 (5.71)		197 (17.84)		165 (14.96)		172 (13.27)		484 (37.35)		458 (35.34)	
	Graduate from primary school	146 (13.22)		291 (26.36)		266 (24.12)		213 (16.44)		316 (24.38)		265 (20.45)	
	Lower middle school degree	380 (34.42)		373 (33.79)		384 (34.81)		407 (31.4)		315 (24.31)		344 (26.54)	
	Upper middle school degree or above	515 (46.65)		243 (22.01)		288 (26.11)		504 (38.89)		181 (13.97)		229 (17.67)	
Former or current smoker, n (%)			662 (59.96)		710 (64.31)		682 (61.83)		32 (2.47)		54 (4.17)		60 (4.63)
Former or current drinker, n (%)			707 (64.04)		717 (64.95)		743 (67.36)		159 (12.27)		130 (10.03)		130 (10.03)
**Physical activity level, n (%)**													
	Low	710 (64.31)		376 (34.06)		434 (39.35)		981 (75.69)		543 (41.9)		609 (47)	
	Medium	203 (18.39)		197 (17.84)		213 (19.31)		142 (10.96)		113 (8.72)		165 (12.73)	
	High	191 (17.3)		531 (48.1)		456 (41.34)		173 (13.35)		640 (49.38)		522 (40.28)	
**BMI (kg/m^2^), n (%)**													
	≤18.4	29 (2.63)		97 (8.79)		57 (5.17)		79 (6.1)		92 (7.1)		75 (5.79)	
	18.5-23.9	531 (48.1)		777 (70.38)		709 (64.28)		667 (51.47)		817 (63.04)		806 (62.19)	
	24.0-27.9	422 (38.22)		191 (17.3)		280 (25.39)		394 (30.4)		322 (24.85)		335 (25.85)	
	≥28.0	122 (11.05)		39 (3.53)		57 (5.17)		156 (12.04)		65 (5.02)		80 (6.17)	
MAMC^d^ (cm), median (IQR)			23.1 (20.8-25.3)		22.4 (20.7-24.1)		22.9 (21.3-24.9)		20.5 (18.7-22.5)		20.0 (18.6-21.6)		20.4 (18.7-22.5)
WHR^e^, median (IQR)			0.90 (0.85-0.93)		0.86 (0.82-0.90)		0.87 (0.82-0.91)		0.84 (0.80-0.89)		0.84 (0.79-0.88)		0.82 (0.79-0.87)
Abdominal obesity^f^, n (%)			526 (47.64)		258 (23.37)		319 (28.92)		608 (46.91)		532 (41.05)		440 (33.95)
Hypertension, n (%)			151 (13.68)		27 (2.45)		58 (5.26)		160 (12.35)		52 (4.01)		56 (4.32)
Diabetes, n (%)			50 (4.53)		6 (0.54)		18 (1.63)		34 (2.62)		13 (1)		23 (1.77)
Fractures, n (%)			52 (4.71)		128 (11.59)		117 (10.61)		37 (2.85)		99 (7.64)		135 (10.42)
**OSTA^g^ index level^h^, n (%)**													
	>−1	984 (89.13)		955 (86.5)		995 (90.21)		1088 (83.95)		1028 (79.32)		1055 (81.4)	
	−1 to −4	106 (9.6)		132 (11.96)		100 (9.07)		172 (13.27)		214 (16.51)		201 (15.51)	
	<−4	14 (1.27)		17 (1.54)		8 (0.73)		36 (2.78)		54 (4.17)		40 (3.09)	
Carbohydrate (g/d), median (IQR)			274.3 (201.7-349.4)		390.3 (322.3-467.5)		360.8 (271.7-465.6)		237.7 (175.3-303.4)		332.5 (261.7-405.1)		320.4 (246.6-402.0)
Total fat (g/d), median (IQR)			71.0 (50.4-95.9)		61.2 (41.8-86.0)		72.9 (50.3-98.9)		60.2 (41.7-83.0)		51.4 (34.0-72.8)		66.7 (45.0-89.0)
Protein (g/d), median (IQR)			75.3 (61.4-92.2)		69.6 (57.4-85.0)		75.8 (62.2-92.2)		63.9 (51.6-80.0)		59.0 (48.0-72.0)		65.5 (53.3-80.1)

^a^The data are presented as mean (SD) or median (IQR) for continuous variables and as n (%) for categorical variables.

^b^Q: quintile.

^c^DII: dietary inflammatory index.

^d^MAMC: midarm muscle circumference.

^e^WHR: waist-to-hip ratio.

^f^A man WHR ≥0.90 and a women WHR ≥0.85 is abdominal obesity.

^g^OSTA: Osteoporosis Self-assessment Tool for Asians.

^h^The OSTA index level distinguishes between different degrees of osteoporosis.

### Association of DII With Fractures

The results of the Cox proportional hazards models suggested that the DII was significantly associated with fracture risk in people (Table 2). However, this correlation exists only in women rather than men. For men, after adjusting for covariates, the HRs for quintiles of DII were 1, 0.96 (95% CI 0.66-1.41), 1.05 (95% CI 0.74-1.49), 0.89 (95% CI 0.62-1.26), and 0.94 (95% CI 0.67-1.34; trend: *P*=.62); for women, after adjusting for covariates, the HRs for quintiles of DII were 1, 1.13 (95% CI 0.72-1.79), 1.24 (95% CI 0.83-1.86), 1.51 (95% CI 1.02-2.22), and 1.62 (95% CI 1.10-2.39; trend: *P*=.004). When DII was treated as a continuous variable, it was also significantly associated with fracture risk in women rather than in men. The 2 results were coinciding with those of the man and woman quintiles.

**Table 2:** Hazard ratios (95% CIs) of fractures according to the quintiles (Qs) of the dietary inflammatory index (DII)^a^.

		Qs of DII, hazard ratio (95% CI)						Trend, *P* value^b^		Continuous DII, hazard ratio (95% CI)
		Q1	Q2	Q3	Q4	Q5				
**Men**										
	Model 1^c^	1 (ref^d^)	0.96 (0.66-1.41)	1.04 (0.73-1.46)	0.89 (0.62-1.24)	0.95 (0.67-1.34)	.63		0.99 (0.92-1.05)	
	Model 2^e^	1 (ref)	0.96 (0.66-1.41)	1.05 (0.74-1.49)	0.88 (0.62-1.25)	0.94 (0.67-1.34)	.63		0.99 (0.92-1.05)	
	Model 3^f^	1 (ref)	0.96 (0.66-1.41)	1.05 (0.74-1.49)	0.89 (0.62-1.26)	0.94 (0.67-1.34)	.62		0.99 (0.92-1.05)	
**Women**										
	Model 1^c^	1 (ref)	1.11 (0.71-1.76)	1.17 (0.78-1.75)	1.45 (0.99-2.13)	1.57 (1.07-2.31)	.005		1.12 (1.04-1.20)	
	Model 2^e^	1 (ref)	1.12 (0.71-1.77)	1.21 (0.81-1.80)	1.48 (1.00-2.18)	1.59 (1.08-2.34)	.049		1.12 (1.04-1.20)	
	Model 3^f^	1 (ref)	1.13 (0.72-1.79)	1.24 (0.83-1.86)	1.51 (1.02-2.22)	1.62 (1.10-2.39)	.004		1.12 (1.04-1.21)	

^a^Q1 indicates participants having the lowest DII values, the least proinflammatory level; Q5 indicates participants having the highest DII values, the most proinflammatory level. Data were hazard ratios (95% CIs), calculated using Cox proportional hazards analyses.

^b^*P* value for trend: tests for trends were performed for continuous variables using categorical DII scores by Qs.

^c^Adjusted for age, gender, residence area, highest education level, household income, and marital status.

^d^ref: reference.

^e^Model 1+smoking status, drinking status, physical activity level, BMI, midarm muscle circumference, and waist-hip ratio.

^f^Model 2+hypertension, diabetes, and Osteoporosis Self-assessment Tool for Asians index level.

### RCS Analysis

**Figure 2:**
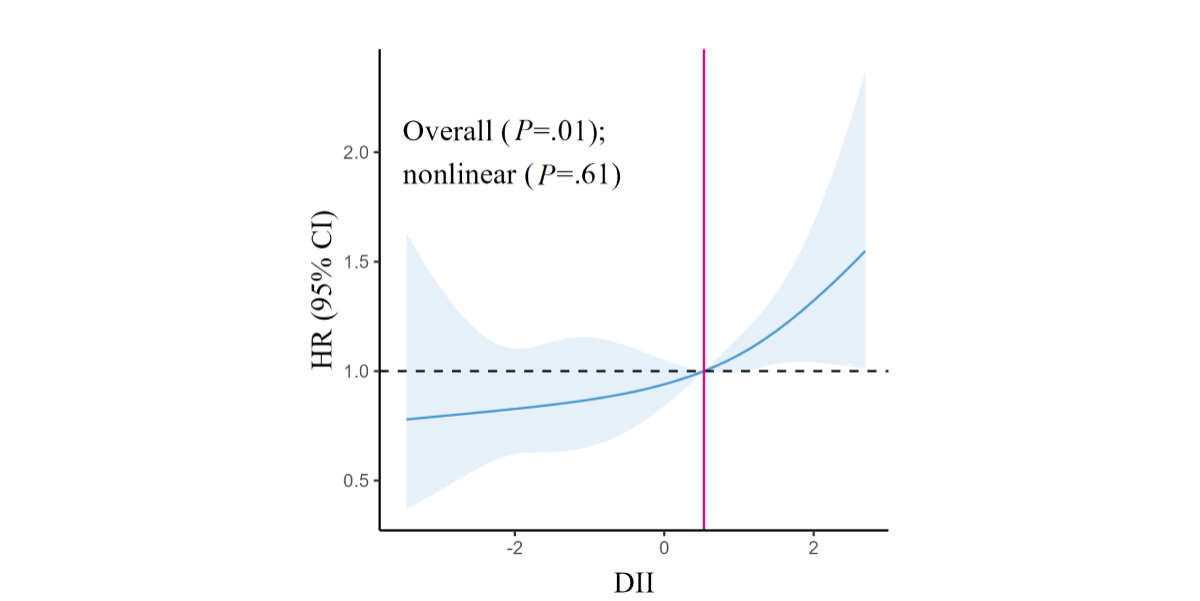
Multivariable-adjusted hazard ratios (HRs; blue solid lines) and 95% CIs (blue shadow) for risk of fracture according to the dietary inflammatory index (DII) score among women in model 3. The median intakes were set as references (black dotted line; HR=1.00). The solid pink line represents the line where the point corresponding to the value of the DII in the curve was located when HR=0.

To further understand the correlation between the DII and fracture risk, we performed an RCS analysis. The RCS analysis showed a significant association between fracture risk and the DII score in women (overall association: *P*=.01; Figure 2). As the DII score was >0.53, HR showed a significant upward trend. However, the spline variable confirmed no significant departure from the nonlinear relationship (nonlinear: *P*=.61) between fracture risk and the DII score.

### Subgroup Analyses

The association between fracture risk and the DII score, as stratified by age, BMI, smoking status, drinking status, obesity status, OSTA index level, and MAMC, was further investigated. Women aged <50 years had a positive association between fracture risk and the DII score (trend: *P*=.003; HR 2.15, 95% CI 1.16-3.97), whereas no association was found among those aged ≥50 years (trend: *P*=.38; HR 1.15, 95% CI 0.69-1.92). Women who are nonsmokers, who are nondrinkers, and with nonabdominal obesity had a positive association between fracture risk and the DII score (trend: *P*=.004; HR 1.64, 95% CI 1.10-2.44; trend: *P*=.02; HR 1.51, 95% CI 1.01-2.27; and trend: *P*=.03; HR 1.92, 95% CI 1.11-3.32, respectively), whereas no association was found among smokers, drinkers, and abdominal obesity (trend: *P*=.37; HR 0.61, 95% CI 0.13-2.97; trend: *P*=.06; HR 3.12, 95% CI 0.86-16.84; and trend: *P*=.07; HR 1.31, 95% CI 0.77-2.23, respectively). Women with an OSTA index level >−1 had a positive association between fracture risk and the DII score (trend: *P*=.001; HR 2.02, 95% CI 1.27-3.21), whereas no association was found among those with an OSTA index level ≤−1 (trend: *P*=.67; HR 0.79, 95% CI 0.40-1.57) and no association was found among those with BMI <24 kg/m^2^, ≥24 kg/m^2^, MAMC <21.46, or MAMC ≥21.46 (Table 3). However, there was no statistically significant association between fracture risk and the DII score in the man subgroup (all *P*>.05; Table S2 in Multimedia Appendix 1). When DII was treated as a continuous variable, these subgroup analyses results were coinciding with the men and women quintiles, which are also shown in the forest plots in Figure S2 in Multimedia Appendix 1. None of the subgroup analyses had interactions (Table 3).

**Table 3:** Hazard ratios (95% CIs) of fractures according to the quintiles (Qs) of the dietary inflammatory index (DII) by age, BMI, smoking status, drinking status, obesity status, Osteoporosis Self-assessment Tool for Asians (OSTA) index level, and midarm muscle circumference (MAMC) in women^a^.

Subgroups		Qs of DII in women, hazard ratio (95% CI)						Trend, *P* value^b^		Interaction, *P* value^c^			Continuous DII, hazard ratio (95% CI)	
		Q1	Q2	Q3	Q4	Q5								
**Age (years)**											.50			
	<50	1 (ref^d^)	1.15 (0.55-2.40)	1.38 (0.73-2.61)	1.62 (0.86-3.01)	2.15 (1.16-3.97)	.003					1.19 (1.06-1.34)		
	≥50	1 (ref)	1.02 (0.57-1.81)	0.90 (0.52-1.54)	1.16(0.70-1.94)	1.15 (0.69-1.92)	.38					1.05 (0.92-1.16)		
**BMI (kg/m^2^)**											.07			
	<24	1 (ref)	1.08 (0.59-1.97)	1.36 (0.79-2.32)	1.45 (0.87-2.44)	1.64 (0.92-2.77)	.03					1.11 (1.01-1.23)		
	≥24	1 (ref)	0.89 (0.45-1.76)	1.14 (0.63-2.07)	1.29 (0.73-2.28)	1.35 (0.76-2.37)	.13					1.11 (1.00-1.24)		
**Smoking status**											.60			
	Former or current smoker	1 (ref)	1.10 (0.28-4.91)	0.74 (0.18-3.46)	0.61 (0.14-2.88)	0.61 (0.13-2.97)	.37					0.88 (0.64-1.24)		
	Nonsmoker	1 (ref)	1.12 (0.70-1.78)	1.23 (0.81-1.86)	1.45 (0.97-2.17)	1.64 (1.10-2.44)	.004					1.13 (1.05-1.22)		
**Drinking status**											.37			
	Former or current drinker	1 (ref)	1.12 (0.17-7.31)	2.61 (0.68-14.40)	2.20 (0.59-12.05)	3.12 (0.86-16.84)	.06					1.26 (0.99-1.65)		
	Nondrinker	1 (ref)	1.12 (0.70-1.79)	1.13 (0.75-1.73)	1.42 (0.95-2.12)	1.51 (1.01-2.27)	.02					1.10 (1.02-1.19)		
**OSTA^e^ index level**											.29			
	>−1	1 (ref)	0.98 (0.55-1.76)	1.44 (0.89-2.34)	1.67 (1.05-2.67)	2.02 (1.27-3.21)	.001					1.20 (1.09-1.31)		
	≤−1	1 (ref)	0.80 (0.38-1.67)	1.14 (0.59-2.21)	0.84 (0.43-1.65)	0.79 (0.40-1.57)	.67					0.96 (0.84-1.10)		
**Obesity status^f^**											.36			
	Abdominal obesity	1 (ref)	0.98 (0.51-1.88)	0.77 (0.42-1.40)	1.23 (0.71-2.10)	1.31 (0.77-2.23)	.07					1.09 (0.98-1.21)		
	Nonabdominal obesity	1 (ref)	1.27 (0.69-2.34)	1.79 (1.03-3.11)	1.30 (0.74-2.29)	1.92 (1.11-3.32)	.03					1.13 (1.02-1.26)		
**MAMC**											.72			
	<21.46	1 (ref)	0.81 (0.51-1.27)	1.16 (0.79-1.70)	1.06 (0.73-1.55)	1.25 (0.86-1.82)	.07					1.07 (0.99-1.15)		
	≥21.46	1 (ref)	1.07 (0.66-1.73)	1.34 (0.52-3.44)	1.38 (0.43-4.43)	1.51 (0.39-5.83)	.54					1.04 (0.97-1.11)		

^a^Values were multivariable-adjusted hazard ratios (95% CIs) for risk of fractures according to Qs of the DII stratified by age, BMI, smoking status, drinking status, obesity status, OSTA index level, and MAMC in model 3. Q1 indicates participants having the lowest DII values, the least proinflammatory level; Q5 indicates participants having the highest DII values, the most proinflammatory level.

^b^*P* value for trend: tests for trends were performed for continuous variables using categorical DII score by Qs.

^c^*P* value for interaction was calculated by contrasting the coefficients of the cross-product of stratified values and DII Qs in the model.

^d^ref: reference.

^e^The OSTA index level distinguishes different degrees of osteoporosis.

^f^A man waist-to-hip ratio ≥0.90 and a woman waist-to-hip ratio ≥0.85 is abdominal obesity.

### RCS Analysis of Subgroup

**Figure 3:**
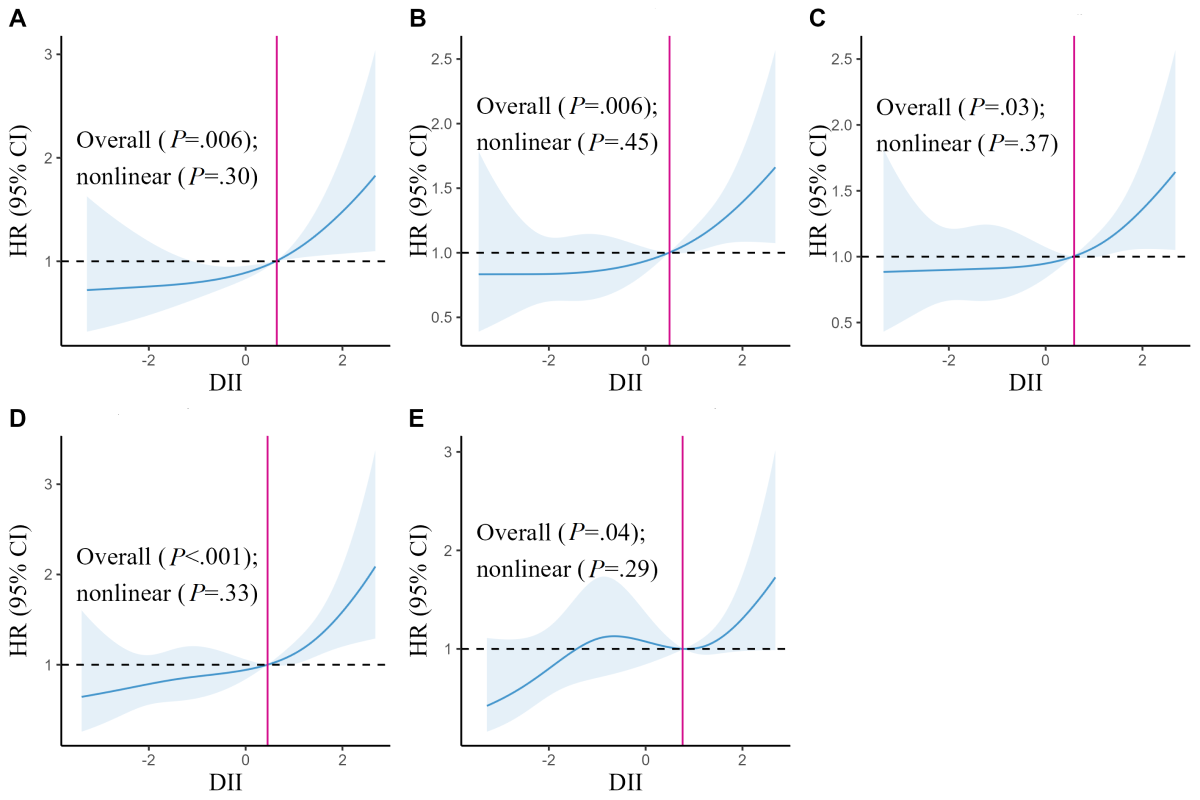
Multivariable-adjusted hazard ratios (HRs; blue solid lines) and 95% CIs (blue shadow) for risk of fracture according to the dietary inflammatory index (DII) score among (A) women with age <50 years, (B) nonsmoker, (C) nondrinker, (D) Osteoporosis Self-assessment Tool for Asians (OSTA) index level >−1, and (E) nonabdominal obesity in model 3. The median intakes were set as references (black dotted line; HR=1.00). The solid pink line represents the line where the point corresponding to the value of the DII in the curve was located when HR=0.

To further understand the correlation between the DII and fracture risk among women in the subgroup, we performed an RCS analysis. We showed the statistically significant subgroups in the Cox regression results of subgroup analysis according to subgroup analysis in Table 3, such as age <50 years (trend: *P*=.003), nonsmoker (trend: *P*=.004), nondrinker (trend: *P*=.02), OSTA index level >−1 (trend: *P*=.001), and nonabdominal obesity (trend: *P*=.03). The RCS analysis results showed a significant association between fracture risk and the DII score among women in the subgroup for age <50 years (overall association: *P*=.006; Figure 3A); nonsmoker (overall association: *P*=.006; Figure 3B); nondrinker (overall association: *P*=.03; Figure 3C); OSTA index level >−1 (overall association: *P*<.001; Figure 3D), and nonabdominal obesity (overall association: *P*=.04; Figure 3E). The results of the RCS analysis of the remaining subgroups are presented in Figure S3 in Multimedia Appendix 1. In general, the curves for age <50 years (Figure 3A), nonsmoker (Figure 3B), nondrinker (Figure 3C), and OSTA index level >−1 (Figure 3D), except for nonabdominal obesity (Figure 3E), showed a trend of rapid rise after a slow rise, and the corresponding nodes were 0.64, 0; 0.49, 0; 0.59, 0; and 0.45, 0; respectively. The curve corresponding to nonabdominal obesity (Figure 3E) also showed a rapid upward trend at node 0.76, 0. These results suggest that the higher the inflammation score of the diet, the greater the risk of fracture and that the cumulative effect of inflammatory foods greatly increases the risk of fractures.

### Sensitivity Analyses

In sensitivity analyses (Table 4), after excluding people with diabetes or hypertension, there was still a positive association between fracture risk and the DII score in women. For women with nonhypertension, the HRs for quintiles of the DII were 1, 0.99 (95% CI 0.59-1.68), 1.44 (95% CI 0.93-2.24), 1.75 (95% CI 1.14-2.68), and 1.91 (95% CI 1.25-2.92; trend: *P*<.001). For women with nondiabetes, the HRs for quintiles of DII were 1, 1.09 (95% CI 0.68-1.74), 1.24 (95% CI 0.82-1.87), 1.50 (95% CI 1.03-2.22), and 1.62 (95% CI 1.09-2.40; trend: *P*=.003). For women with nonhypertension and diabetes, the HRs for quintiles of the DII were 1, 1.16 (95% CI 0.68-1.99), 1.42 (95% CI 0.90-2.25), 1.78 (95% CI 1.15-2.78), and 1.97 (95% CI 1.26-3.06; trend: *P*<.001). For women with nonhypertension or diabetes, the HRs for quintiles of the DII were 1, 1.09 (95% CI 0.69-1.73), 1.20 (95% CI 0.80-1.79), 1.46 (95% CI 0.99-2.14), and 1.56 (95% CI 1.06-2.30; trend: *P*=.006). When the DII was treated as a continuous variable, it was significantly associated with fracture risk in women with nonhypertension, nondiabetes, nonhypertension and diabetes, and nonhypertension or diabetes. These results are consistent with those of the quintiles.

**Table 4:** Sensitivity analyses of dietary inflammatory index (DII) in association with fracture risk in women^a^.

Groups	Qs^b^ of DII in women, hazard ratio (95% CI)						Trend, *P* value^c^		Continuous DII, hazard ratio (95% CI)
	Q1	Q2	Q3	Q4	Q5				
Nonhypertension	1 (ref^d^)	0.99 (0.59-1.68)	1.44 (0.93-2.24)	1.75 (1.14-2.68)	1.91 (1.25-2.92)	<.001		1.19 (1.06-1.34)	
Nondiabetes	1 (ref)	1.09 (0.68-1.74)	1.24 (0.82-1.87)	1.50 (1.03-2.22)	1.62 (1.09-2.40)	.003		1.05 (0.92-1.16)	
Nonhypertension and diabetes	1 (ref)	1.16 (0.68-1.99)	1.42 (0.90-2.25)	1.78 (1.15-2.78)	1.97 (1.26-3.06)	<.001		1.11 (1.01-1.23)	
Nonhypertension or diabetes	1 (ref)	1.09 (0.69-1.73)	1.20 (0.80-1.79)	1.46 (0.99-2.14)	1.56 (1.06-2.30)	.006		1.11 (1.00-1.24)	

^a^Values are multivariable-adjusted hazard ratios (95% CIs) for the risk of fractures according to quintiles of the DII stratified by hypertension and diabetes status in model 3. Q1 indicates participants having the lowest DII values, the least proinflammatory level; Q5 indicates participants having the highest DII values, the most proinflammatory level.

^b^Q: quintile.

^c^*P* value for trend: tests for trends were performed for continuous variables using categorical DII scores by Qs.

^d^ref: reference.

### Association of DII Components With Fractures

The association of macronutrients, some micronutrient DII scores, and fracture risk in men and women is shown in Table S3 in Multimedia Appendix 1. The DII scores for protein (trend: *P*=.03), niacin (trend: *P*=.002), and iron (trend: *P*=.02) showed significant associations with fracture risk in women. The higher the intake of protein, the higher the risk of fracture. The higher the intake of niacin and iron, the lower the risk of fracture. The DII scores for carbohydrate, total fat, and protein were associated with fracture risk in men. The association between other macronutrients and micronutrient DII scores and fracture risk is shown in Table S3 in Multimedia Appendix 1.

## Discussion

### Principal Findings

This study, which used a large-scale sample in China, aimed to prospectively explore the association between dietary inflammatory potential and fracture risk. This study found that a higher DII score (more proinflammatory diets) was associated with a higher risk of fracture in women than in men. To confirm this conclusion, we used Cox proportional hazards models to demonstrate a positive correlation between DII and fracture risk in women and used an RCS analysis to further illustrate the trend of fracture risk with the DII score. In addition, we performed subgroup and sensitivity analyses to further verify the stability of the results. This reliable and robust result can provide dietary interventions and strategies for fracture prevention, promote bone health, provide a specific basis for China’s Healthy Lifestyle Action, and promote the Healthy China development agenda.

### Comparison With Prior Work

In this study, the association between the DII and fracture risk differed by gender. Statistically significant results were observed among woman participants rather than man participants. This finding is consistent with previous studies [40,54]. Estrogen is hypothesized to have a protective anti-inflammatory effect [55,56]. Other studies have reported that inflammation has different roles and mechanisms in men and women (eg, for cardiovascular diseases) [57]. In addition, compared with women, men have worse habits, which offsets the positive effect of diet on the prevention of fractures. Tobacco smoke is a proinflammatory agent. It may be that the effect of tobacco swamps the effect of dietary sources of inflammation [58]. In this study, men smoked at about 16 times the rate of women (62.6% of men were past or present smokers vs only about 3.8% of women). However, 2 studies from Guangdong and Hong Kong in China showed an association between the DII and fractures in both genders [23,43]. It may be difficult to directly compare the findings of a case-control study with those of a longitudinal study. In addition, 2 studies considered the older adult population as the research object, and this gender difference became less pronounced with advancing age [59]. We believe that for women, reducing the intake of a proinflammatory diet or increasing the intake of an anti-inflammatory diet, such as fruits, vegetables, and tea, can decrease fracture risk. Consuming ≤5 servings per day of fruit and vegetables is associated with a higher hip fracture risk [60]. Tea and tea extract flavonoids with antioxidant and anti-inflammatory properties have osteoprotective effects on bone biology. Huang et al [61] showed that high tea consumption versus no tea consumption reduced fracture risk by 31%. In postmenopausal women, estrogen levels decrease significantly with age and the protective anti-inflammatory effect is weakened, which can easily cause osteoporosis and increase the risk of fractures.

The RCS analysis showed a changing trend that fracture risk increased with increasing DII scores. Notably, as the DII score was >0.53, HR had shown a significant upward trend. The cumulative effect of inflammatory foods greatly increases the risk of fractures. Therefore, the intake of inflammatory foods should be reduced as early as possible to avoid the accumulation of inflammatory effects of the diet, thereby reducing the risk of fractures.

To our surprise, the research results are present only in women aged <50 years rather than those aged ≥50 years. However, a study of postmenopausal White women confirmed that an inflammatory diet is associated with fractures [32]. This may be due to a significant change in estrogen levels after menopause in women who are prone to osteoporosis and an increase in the risk of fractures [62,63]. However, our results still need to be discussed because of population characteristics, dietary patterns, and differences in the instruments used to assess diet quality. There are 2 possible explanations for this finding. On the one hand, with increasing age, especially in older adults, due to altered body function and physiological and pathological reasons, there is less food intake and food use than in the young. On the other hand, it could be that the benefits of a less inflammatory dietary pattern for bone health are overshadowed by the much greater risk for fracture produced by aging [32,64]. Therefore, our study highlights the importance of early dietary intervention. Health gains are predicted to be larger when earlier dietary changes are initiated in life. Sustained changes from a typical to an optimized diet from an early age could translate into an increase in life expectancy of ≥10 years [65]. In addition, we recommend that women aged ≥50 years should be the focus of the fracture population and take measures to enhance their intake and use of anti-inflammatory diets.

We observed that a less inflammatory diet reduced the risk of fractures in women with nonabdominal obesity. However, this result was not observed in women with abdominal obesity. Therefore, the effect of the regional accumulation of body fat on fractures needs to be considered. Intraabdominal obesity is an important risk factor for low-grade inflammation [66]. A preferential accumulation of visceral adipose tissue and adipose tissue promotes proinflammatory factor production, which drives chronic low-grade inflammation to cause diabetes, cardiovascular disease, and metabolic disease [66-69]. Abdominal obesity is highly prevalent in China [70,71]. Maintaining a good body weight and body shape is essential for the prevention of fractures.

Furthermore, we did not find an association between DII and fractures in women who smoke and drink. Smoking and excessive alcohol consumption induce oxidative stress to weaken the antioxidant effects and activate a variety of transcription factors such as inflammatory cytokines [72]. Smoking affects the intake of some antioxidants in food, such as vitamins C and E, to reduce the effect of an anti-inflammatory diet [73-76]. In addition, if people consume high amounts of alcohol, they may also consume high amounts of carbohydrates and energy, which have a proinflammatory score on the DII [39]. A cohort study in an Italian population showed that a healthy lifestyle, that is, adherence to a healthy dietary pattern, abstinence from smoking and regular engagement in physical activity, and has a positive effect on reducing the risk of all-cause mortality, especially when the combined effect of all 3 lifestyle behaviors was considered [77]. Therefore, we advocate smoking cessation and alcohol restriction and eat more anti-inflammatory foods to maximize the positive effect of diet on fracture prevention.

In addition, our study found that the DII scores of protein, niacin, and iron showed significant associations with fracture risk in women. Protein intake increases the fracture risk in women. Excessive protein intake can lead to dietary acid load, and an acidic diet can lead to osteoporosis and an increased risk of fractures [78,79]. However, recent systematic reviews have generally stated that protein intake reduces fracture risk [60]. Wallace et al [80] showed that a high dietary protein intake was associated with a 16% reduction in hip fracture risk compared with a low protein intake. In addition, Rizzoli et al [60] showed that inadequate protein intake in the diet may cause more disease problems than excessive protein intake. The niacin intake reduced the fracture risk in women. The potential beneficial mechanisms of action of niacin on bone are through decreased inflammation. Niacin decreases CRP levels, and higher CRP levels are associated with fractures [81]. B-vitamins appear to influence the development of collagen and alter the metabolism of osteoblasts in a dose-dependent manner [82,83]. Carbone et al [84] showed that dietary niacin intake was significantly associated with an increased risk of hip fracture per HRs (95% CI 1.01-1.24) with spline models, suggesting a U-shaped association. Clinical studies have shown that the incidence of osteoporosis and fractures in iron metabolism disorders is significantly increased and that iron deficiency affects bone metabolism. In healthy menopausal women, dietary iron is positively correlated with bone mineral density and may decrease the risk of fractures [85]. However, excess iron increases oxidative stress, causing inflammatory changes that destroy bones. Therefore, the relationship between iron and fracture needs further research [86]. It is worth noting that the intake of macronutrients (carbohydrates, total fat, and protein) was associated with fracture risk in men. However, this result was not observed when considering the overall diet. Therefore, it is reasonable to believe that a single nutrient does not have a significant impact on fracture risk. Because of the complexity of the interactions between diets, we believe that overall dietary optimization is an important way to improve bone health.

### Strengths and Limitations

Our study has several strengths. First, we used a large-scale, nationally representative, prospective cohort design study to examine the association with dietary inflammatory potential and fractures. Second, we controlled for multiple confounding factors and performed subgroup and sensitivity analyses to ensure that the results were robust and reliable. Our results can provide data support for subsequent studies. Third, our research results, which demonstrated the relationship between diet and fracture risk in women aged 18 to 49 years, can supplement diet and fracture relationships among low age groups.

There are several limitations to this study that should be considered in with regard to its results. First, possible confounding variables existed and could have affected the outcome. There may be variables that can affect fractures such as osteoporosis or bone mineral density. Second, the fractures were self-reported and not clinically confirmed. This study did not distinguish between the cause and specific site of the fracture. Third, this study did not consider the probability of patients being lost to follow-up due to the fractures themselves, which may underestimate the incidence of fractures. Fourth, the correlation between the DII and fracture risk varies between men and women, which may be due to the incompatibility of the mechanisms of action of inflammatory effects in men and women, and further research is needed. Whether lowering the DII score in the future reduces the risk of fractures will require higher-quality randomized controlled trials to be validated.

### Conclusions

In conclusion, proinflammatory diet consumption increased the fracture risk in Chinese women aged <50 years. The high consumption of anti-inflammatory foods and low consumption of proinflammatory foods may be an important strategy to prevent fractures in women. Future randomized controlled trials with diets rich in anti-inflammatory components are needed to confirm causality and to consider whether such interventions can reduce the incidence of fractures.

## Appendix

Multimedia Appendix 1 Data tables and figures used to support the findings reported in the paper.

## Abbreviations

CHNS: China Health and Nutrition Survey
CRP: C-reactive protein
DII: dietary inflammatory index
HR: hazard ratio
IL: interleukin
MAMC: midarm muscle circumference
OSTA: Osteoporosis Self-assessment Tool for Asians
PAL: physical activity level
RCS: restricted cubic spline
TNF: tumor necrosis factor
WHR: waist-to-hip ratio

## Data Availability

The data that support the findings of this study are available from the China Health and Nutrition Survey [87].
