# Supplementary material for: Association Between the Dietary Inflammatory Index and the Risk of Fracture in Chinese Adults: Longitudinal Study
Source: JMIR Public Health Surveill. 2023 Aug 17;9:e43501. doi: 10.2196/43501 (PMC10472179; doi:10.2196/43501)
Supplement: Multimedia Appendix 1 [file publichealth_v9i1e43501_app1.docx]

**Table S1.** Baseline characteristics of participants by gender ^a^.

| Characteristics | | Men | Women | *P* value ^b^ | Total |
| --- | --- | --- | --- | --- | --- |
| **Age (years), mean (SD)** | | 44.5 (14.6) | 43.6 (14.5) | .001 | 44.0 (14.6) |
| **DII ^c^, median (IQR)** | | 0.75 (-1.68-1.50) | 0.53 (-1.79-1.42) | <.001 | 0.64 (-1.74-1.46) |
| **Marital status, n (%)** | | | | <.001 |  |
|  | Never married | 658 (11.92) | 325 (5.02) |  | 983 (8.19) |
|  | Married | 4682 (84.83) | 5627 (86.84) |  | 10,309 (85.92) |
|  | Divorced | 65 (1.18) | 82 (1.27) |  | 147 (1.23) |
|  | Widowed | 114 (2.07) | 446 (6.88) |  | 560 (4.67) |
| **Residence, n (%)** | | | | .45 |  |
|  | Rural | 3334 (60.41) | 3959 (61.1) |  | 7293 (60.78) |
|  | Urban | 2185 (39.59) | 2521 (38.9) |  | 4706 (39.22) |
| **Household income level (yuan ¥; yuan ¥1=US $0.14), n (%)** | | | | .86 |  |
|  | Low (<8532) | 1371 (24.84) | 1630 (25.15) |  | 3001 (25.01) |
|  | Medium (8532-15,576) | 1381 (25.03) | 1618 (24.97) |  | 2999 (24.99) |
|  | High (15,577-30,500) | 1371 (24.84) | 1634 (25.22) |  | 3005 (25.04) |
|  | Very high (>30,500) | 1396 (25.29) | 1598 (24.66) |  | 2994 (24.95) |
| **Education level, n (%)** | | | | <.001 |  |
|  | None | 737 (13.35) | 1800 (27.78) |  | 2537 (21.14) |
|  | Graduate from primary school | 1139 (20.63) | 1299 (20.05) |  | 2438 (20.32) |
|  | Lower middle school degree | 1903 (34.48) | 1832 (28.27) |  | 3735 (31.13) |
|  | Upper middle school degree or above | 1740 (31.53) | 1549 (23.9) |  | 3289 (27.41) |
| **Former or current smoker, n (%)** | | 3457 (62.64) | 247 (3.81) | <.001 | 3704 (30.87) |
| **Former or current drinker, n (%)** | | 3520 (63.78) | 682 (10.52) | <.001 | 4202 (35.02) |
| **Physical activity level, n (%)** | | | | <.001 |  |
|  | Low | 2535 (45.93) | 3637 (56.13) |  | 6172 (51.44) |
|  | Medium | 991 (17.96) | 721 (11.13) |  | 1712 (14.27) |
|  | High | 1993 (36.11) | 2122 (32.75) |  | 4115 (34.29) |
| **BMI (kg/m^2^), n (%)** | | | | <.001 |  |
|  | ≤18.4 | 279 (5.06) | 422 (6.51) |  | 701 (5.84) |
|  | 18.5-23.9 | 3385 (61.33) | 3775 (58.26) |  | 7160 (59.67) |
|  | 24.0-27.9 | 1471 (26.65) | 1753 (27.05) |  | 3224 (26.87) |
|  | ≥28.0 | 384 (6.96) | 530 (8.18) |  | 914 (7.62) |
| **MAMC ^d^(cm), median (IQR)** | | 22.8 (20.9-24.8) | 20.4 (18.6-22.2) | <.001 | 21.5 (19.4-23.7) |
| **WHR ^e^, median (IQR)** | | 0.87 (0.83-0.92) | 0.84 (0.79-0.88) | <.001 | 0.85 (0.81-0.90) |
| **Abdominal obesity ^f^, n (%)** | | 1884 (34.14) | 2723 (42.02) | <.001 | 4607 (38.39) |
| **Hypertension, n (%)** | | 402 (7.28) | 471 (7.27) | .97 | 873 (7.28) |
| **Diabetes, n (%)** | | 126 (2.28) | 128 (1.98) | .24 | 254 (2.12) |
| **OSTA ^g^ index level ^h^, n (%)** | | | | <.001 |  |
|  | >−1 | 4857 (88.01) | 5267 (81.28) |  | 10,124 (84.37) |
|  | −1 to −4 | 588 (10.65) | 984 (15.19) |  | 1572 (13.1) |
|  | <−4 | 74 (1.34) | 229 (3.53) |  | 303 (2.53) |
| **Carbohydrate (g/d), median (IQR)** | | 330.7 (246.5-423.5) | 282.8 (212.1-362.8) | <.001 | 305.5 (227.2-391.0) |
| **Total fat (g/d), median (IQR)** | | 68.9 (46.8-94.6) | 59.8 (40.5-83.3) | <.001 | 63.6 (42.9-88.5) |
| **Protein (g/d), median (IQR)** | | 70.9 (57.4-86.6) | 60.1 (48.4-74.4) | <.001 | 65.0 (51.8-80.5) |

^a^ The data are presented as mean (SD) or median (IQR) for continuous variables and as n (%) for categorical variables.

^b^ *P* value were based on the differences in characteristics between men and women and were calculated from chi-square tests for categorical variables and rank-sum tests for continuous variables.

^c^ DII: dietary inflammatory index.

^d^ MAMC: midarm muscle circumference.

^e^ WHR: waist-to-hip ratio.

^f^ A man WHR ≥0.90 and a woman WHR ≥0.85 is abdominal obesity.

^g^ OSTA: Osteoporosis Self-assessment Tool for Asians.

^h^ The OSTA index level distinguishes between different degrees of osteoporosis.

**Table S2.** Hazard ratios (95% CIs) of fractures according to the quintiles (Qs) of the dietary inflammatory index (DII) by age, BMI, smoking status, drinking status, obesity status, Osteoporosis Self-assessment Tool for Asians (OSTA) index level, and midarm muscle circumference (MAMC) in men ^a^.

| Subgroups | | Qs of DII in men, hazard ratio (95% CI) | | | | | Trend, *P* value ^b^ | Interaction, *P* value ^c^ | Continuous DII, hazard ratio (95% CI) |
| --- | --- | --- | --- | --- | --- | --- | --- | --- | --- |
|  | | Q1 | Q2 | Q3 | Q4 | Q5 |  |  |  |
| **Age (years)** | | | | | | | | .96 |  |
|  | <50 | 1 (ref^d^) | 1.12 (0.71-1.77) | 0.88 (0.56-1.39) | 0.92 (0.58-1.44) | 0.86 (0.54-1.35) | .21 |  | 0.96  (0.88-1.05) |
|  | ≥50 | 1 (ref) | 1.14 (0.60-2.18) | 1.17 (0.65-2.13) | 0.99 (0.55-1.78) | 1.17 (0.66-2.08) | .86 |  | 1.01  (0.91-1.13) |
| **BMI (kg/m^2^)** | | | | | | | | .65 |  |
|  | <24 | 1 (ref) | 1.06 (0.69-1.62) | 0.75 (0.49-1.15) | 0.87 (0.58-1.32) | 0.81 (0.54-1.24) | .16 |  | 0.95  (0.87-1.04) |
|  | ≥24 | 1 (ref) | 0.67 (0.32-1.43) | 1.06 (0.57-1.99) | 1.28 (0.71-2.29) | 1.02 (0.56-1.85) | .39 |  | 1.05  (0.94-1.18) |
| **Smoking status** | | | | | | | | .24 |  |
|  | Former or current smoker | 1 (ref) | 0.82 (0.50-1.33) | 0.91 (0.59-1.41) | 0.66 (0.42-1.03) | 0.87 (0.56-1.34) | .40 |  | 0.96  (0.88-1.05) |
|  | Nonsmoker | 1 (ref) | 1.68 (0.90-3.14) | 1.36 (0.74-2.52) | 1.49 (0.82-2.70) | 1.28 (0.67-2.26) | .89 |  | 1.02  (0.92-1.14) |
| **Drinking status** | | | | | | | | .09 |  |
|  | Former or current drinker | 1 (ref) | 1.08 (0.65-1.78) | 1.12 (0.70-1.79) | 1.17 (0.74-1.85) | 1.02 (0.64-1.63) | .84 |  | 1.02  (0.93-1.12) |
|  | Nondrinker | 1 (ref) | 0.94 (0.52-1.69) | 0.94 (0.54-1.61) | 0.56 (0.32-0.99) | 0.83 (0.49-1.40) | .20 |  | 0.93  (0.84-1.04) |
| **OSTA ^e^ index level** | | | | | | | | .89 |  |
|  | >−1 | 1 (ref) | 1.00 (0.67-1.49) | 1.03 (0.71-1.50) | 0.92 (0.64-1.34) | 0.88 (0.61-1.27) | .42 |  | 0.98  (0.91-1.05) |
|  | ≤−1 | 1 (ref) | 1.58 (0.46-5.40) | 1.54 (0.48-4.94) | 1.00 (0.30-3.34) | 1.62 (0.51-5.12) | .81 |  | 1.07  (0.86-1.33) |
| **Obesity status ^f^** | | | | | | | | .29 |  |
|  | Abdominal obesity | 1 (ref) | 1.14 (0.72-1.81) | 0.91 (0.58-1.44) | 1.13 (0.72-1.76) | 1.07 (0.68-1.67) | .90 |  | 0.98  (0.88-1.10) |
|  | Nonabdominal obesity | 1 (ref) | 1.18 (0.61-2.31) | 1.43 (0.78-2.62) | 1.24 (0.68-2.25) | 0.89 (0.48-1.64) | .40 |  | 0.99  (0.90-1.08) |
| **MAMC** | | | | | | | | .32 |  |
|  | <21.46 | 1 (ref) | 1.02 (0.51-2.06) | 1.01 (0.53-1.95) | 0.55 (0.28-1.10) | 0.78 (0.41-1.52) | .14 |  | 0.93  (0.81-1.06) |
|  | ≥21.46 | 1 (ref) | 1.04 (0.66-1.63) | 0.99 (0.65-1.50) | 1.01 (0.66-1.52) | 0.99 (0.66-1.50) | .90 |  | 1.00  (0.93-1.09) |

^a^Values were multivariable-adjusted hazard ratios (95% CIs) for risk of fractures according to Qs of the DII stratified by age, BMI, smoking status, drinking status, obesity status, OSTA index level, and MAMC in model 3. Q1 indicates participants having the lowest DII values, the least proinflammatory level; Q5 indicates participants having the highest DII values, the most proinflammatory level.

^b^*P* value for trend: tests for trends were performed for continuous variables using categorical DII score by Qs.

^c^*P* value for interaction was calculated by contrasting the coefficients of the cross-product of stratified values and DII Qs in the model.

^d^ref: reference.

^e^The OSTA index level distinguishes different degrees of osteoporosis.

^f^A man waist-to-hip ratio ≥0.90 and a woman waist-to-hip ratio ≥0.85 is abdominal obesity.

**Figure S1.** A histogram of the quintiles of the dietary inflammatory index (DII) in men and women. (A) DII in men, (B) DII <0 in men, (C) DII >0 in men, (D) DII in women, (E) DII <0 in women, and (F) DII >0 in women.

**
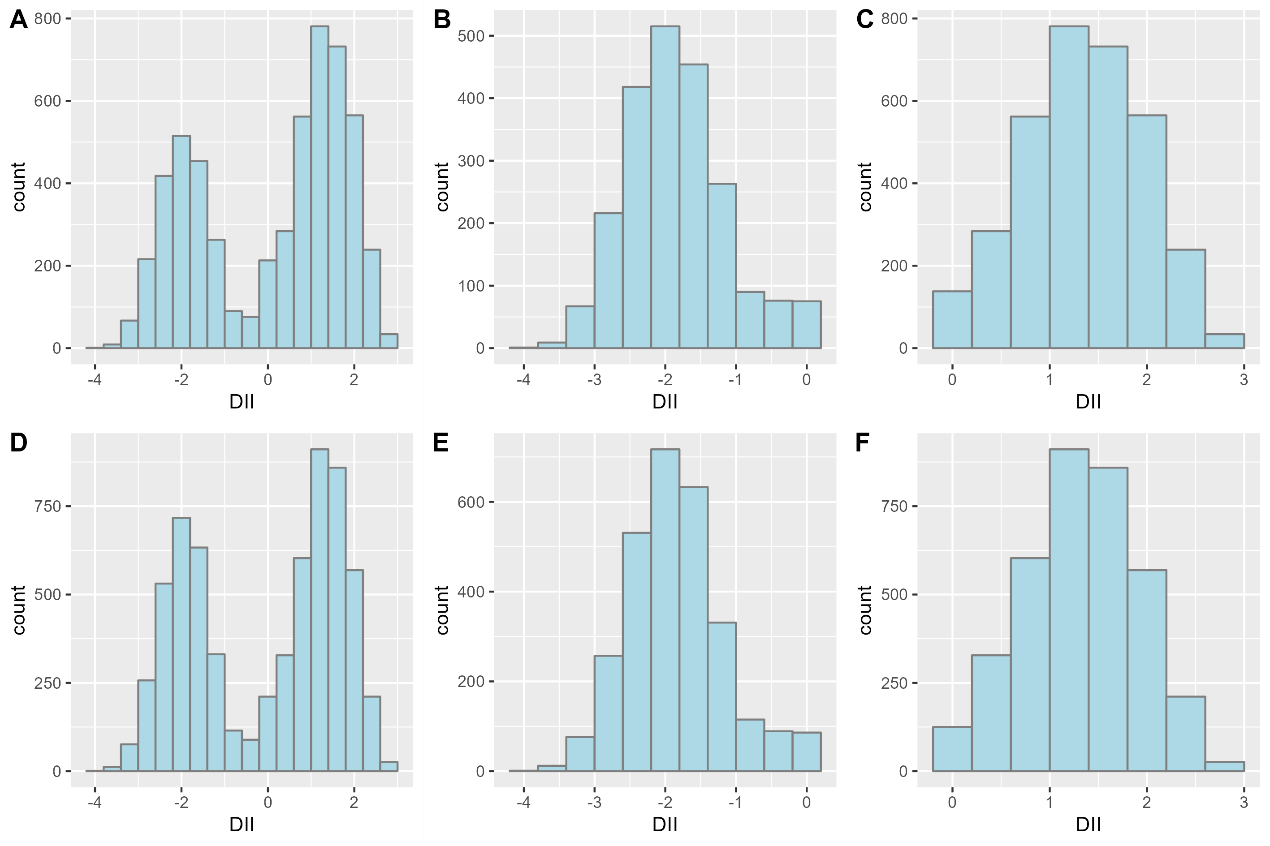
**

**Figure S2.** Hazard ratios (95% CIs) of fractures according to the quintiles of the dietary inflammatory index (DII) by age, BMI, smoking status, drinking status, obesity status, Osteoporosis Self-assessment Tool for Asians (OSTA) index level, and midarm muscle circumference (MAMC) in (A) men and (B) women.

**Figure S3.** Multivariable-adjusted hazard ratios (HRs; blue solid lines) and 95% CIs (blue shadow) for risk of fracture according to the dietary inflammatory index (DII) score among (A) women with age ≥50 years, (B) BMI <24 kg/m^2^, (C) BMI ≥24 kg/m^2^, (D) former or current smoker, (E) former or current drinker, (F) Osteoporosis Self-assessment Tool for Asians (OSTA) index level >−1, (G) nonabdominal obesity, (H) MAMC <21.46, and (I) MAMC ≥21.46 in model 3. The median intakes were set as references (black dotted line; HR=1.00). The solid pink line represents the line where the point corresponding to the value of the DII in the curve was located when HR=0.


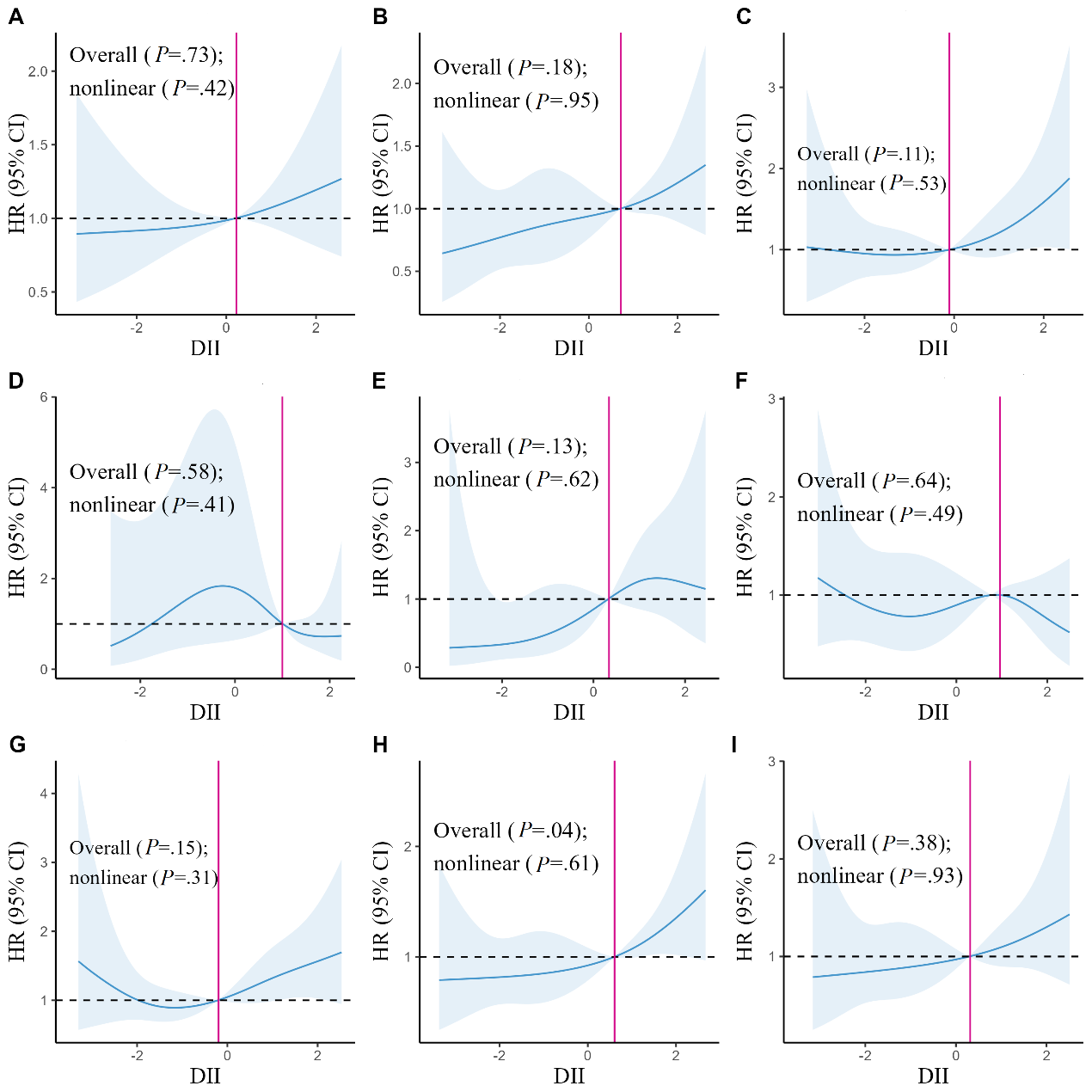


**Table S3.** Hazard Ratios (95% CIs) of fractures according to the quintiles (Qs) of the dietary inflammatory index (DII) components in men and women ^a^.

|  | | Qs^b^ of DII component, hazard ratio (95% CI) | | | | | Trend, *P* value ^c^ |
| --- | --- | --- | --- | --- | --- | --- | --- |
|  | | Q1 | Q2 | Q3 | Q4 | Q5 |  |
| **Men** | | | | | | | |
| **Macronutrients** | | | | | | | |
|  | Carbohydrate | 1 (ref ^d^) | 1.03 (0.83-1.28) | 0.90 (0.72-1.12) | 0.89 (0.72-1.11) | 0.76 (0.60-0.97) | .009 |
|  | Total fat | 1 (ref) | 1.09 (0.89-1.33) | 1.08 (0.87-1.33) | 1.29 (1.04-1.59) | 1.22 (0.98-1.54) | .02 |
|  | Protein | 1 (ref) | 1.47 (1.20-1.80) | 1.17 (0.94-1.45) | 1.38 (1.11-1.71) | 1.33 (1.05-1.69) | .002 |
| **Micronutrients** | | | | | | | |
|  | Vitamin A | 1 (ref) | 1.06 (0.79-1.42) | 1.20 (0.91-1.58) | 1.32 (1.02-1.73) | 1.06 (0.81-1.40) | .52 |
|  | Thiamin | 1 (ref) | 0.76 (0.56-1.03) | 1.04 (0.88-1.34) | 1.09 (0.85-1.40) | 1.10 (0.86-1.41) | .10 |
|  | Riboflavin | 1 (ref) | 0.86 (0.66-1.14) | 0.87 (0.67-1.12) | 1.06 (0.88-1.27) | 1.09 (0.91-1.31) | .23 |
|  | Niacin | 1 (ref) | 0.94 (0.70-1.27) | 1.11 (0.85-1.45) | 1.06 (0.81-1.39) | 1.38 (1.06-1.79) | .002 |
|  | Folic acid | 1 (ref) | 0.68 (0.49-0.93) | 0.98 (0.77-1.26) | 1.07 (0.83-1.36) | 1.18 (0.92-1.50) | .007 |
|  | iron | 1 (ref) | 1.04 (0.87-1.25) | 1.02 (0.85-1.22) | 0.79 (0.61-1.03) | 0.86 (0.66-1.12) | .14 |
|  | selenium | 1 (ref) | 0.85 (0.63-1.16) | 1.11 (0.85-1.44) | 1.17 (0.90-1.52) | 1.05 (0.81-1.37) | .32 |
|  | zinc | 1 (ref) | 0.96 (0.71-1.30) | 1.18 (0.90-1.55) | 1.29 (1.00-1.68) | 1.09 (0.83-1.43) | .32 |
|  | magnesium | 1 (ref) | 0.77 (0.57-1.05) | 1.13 (0.87-1.46) | 1.12 (0.88-1.44) | 1.08 (0.83-1.39) | .23 |
| **Women** | | | | | | | |
| **Macronutrients** | | | | | | | |
|  | Carbohydrate | 1 (ref) | 0.77 (0.57-1.05) | 0.87 (0.65-1.17) | 0.65 (0.48-0.90) | 0.64 (0.46-0.89) | .007 |
|  | Total fat | 1 (ref) | 0.98 (0.73-1.31) | 0.99 (0.73-1.34) | 1.30 (0.97-1.76) | 1.16 (0.84-1.60) | .10 |
|  | Protein | 1 (ref) | 1.51 (1.12-2.03) | 1.35 (0.99-1.84) | 1.41 (1.03-1.94) | 1.61 (1.15-2.25) | .03 |
| **Micronutrients** | | | | | | | |
|  | Vitamin A | 1 (ref) | 1.15 (0.74-1.79) | 1.33 (0.88-2.00) | 1.43 (0.93-2.13) | 1.48 (0.99-2.22) | .03 |
|  | Thiamin | 1 (ref) | 0.77 (0.49-1.22) | 1.05 (0.72-1.54) | 1.14 (0.79-1.65) | 1.34 (0.93-1.92) | .01 |
|  | Riboflavin | 1 (ref) | 0.84 (0.53-1.34) | 1.16 (0.78-1.74) | 1.22 (0.84-1.79) | 1.40 (0.95-2.06) | .01 |
|  | Niacin | 1 (ref) | 0.68 (0.43-1.11) | 1.20 (0.81-1.77) | 1.16 (0.78-1.72) | 1.49 (1.02-2.19) | .002 |
|  | Folic acid | 1 (ref) | 0.69 (0.43-1.11) | 1.05 (0.73-1.52) | 1.22 (0.86-1.75) | 1.25 (0.87-1.79) | .03 |
|  | iron | 1 (ref) | 0.86 (0.67-1.10) | 0.84 (0.65-1.08) | 0.54 (0.37-0.84) | 0.75 (0.52-1.09) | .02 |
|  | selenium | 1 (ref) | 0.64 (0.40-1.02) | 1.15 (0.80-1.67) | 1.08 (0.75-1.57) | 1.06 (0.73-1.54) | .42 |
|  | zinc | 1 (ref) | 0.88 (0.56-1.38) | 1.16 (0.78-1.72) | 1.25 (0.85-1.82) | 1.38 (0.94-2.02) | .02 |
|  | magnesium | 1 (ref) | 0.61 (0.38-0.98) | 1.05 (0.72-1.53) | 1.05 (0.73-1.50) | 1.26 (0.87-1.81) | .02 |

^a^ Values are multivariable-adjusted hazard ratios (95% CIs) for the risk of fractures according to quintiles of the DII component in model 3. Q1 indicates participants having the lowest DII values, the least proinflammatory level; Q5 indicates participants having the highest DII values, the most proinflammatory level.

^b^ Q: quintile.

^c^ *P* value for trend: tests for trends were performed for continuous variables using categorical DII scores by Qs.

^d^ ref: reference.
